# Transformation of medical libraries: Reflections from a 20-year journey

**DOI:** 10.51866/mol.947

**Published:** 2025-07-14

**Authors:** Apichai Wattanapisit

**Affiliations:** 1 MD, Dip. Thai Board of Family Medicine, Academic Fellowship (Family Medicine), Department of Clinical Medicine, School of Medicine Walailak University, Nakhon Si Thammarat, Thailand.; 2 Family Medicine Clinic, Walailak University Hospital, Nakhon Si Thammarat, Thailand.; 3 The Excellent Center of Community Health Promotion, Walailak University, Nakhon Si Thammarat, Thailand. Email: apichai.wa@wu.ac.th

**Keywords:** Digital media, Digital technology, Medical library

Twenty years ago, I walked into a medical school in Thailand as a young student. It was a new world filled with new experiences. One of the most fascinating places was the medical library - a dedicated space within the faculty of medicine where I could find books and other learning materials. As a student, I often visited the library to study and read. Although the building was not large, it offered a wide selection of books and journals across medical and related fields. Electronic materials were available even then, but physical resources still dominated.

Last month (April 2025), I returned to that same medical library. The transformation was striking. Physical books and journals occupied less than a quarter of the space they once did. In their place were open study zones and shared working areas. At the front desk, two computers provided access to digital databases - housing books, journals and electronic learning materials. The library had clearly shifted from a physical to a digital environment.

I also enjoy visiting public, academic and medical libraries at other institutions. From these visits, I have observed that public libraries still rely heavily on physical books, with patrons reading onsite or borrowing materials for home use. Laptop use is common. In contrast, academic and medical libraries now primarily serve users who bring their own devices, such as laptops, tablets and smartphones. The use of physical books is increasingly becoming rare. I have explored many library websites and found that while digital systems now replace manual catalogue searches, some materials still require in-person access to the shelves. More recent books and journals are published and accessed online.

These observations reflect the broader transformation of medical libraries. This shift has been discussed for a couple of decades. For example, a 2005 perspective in the New England Journal of Medicine described the future of medical libraries in a ‘post-Google world’ as ‘multimedia digital libraries’.^1^ A 2014 publication highlighted the evolving roles and skills of medical librarians.^2^ Purchasing digital databases and electronic resources remains expensive, especially in low- and middle-income countries such as Thailand. The cost-effectiveness of digital libraries is still an ongoing challenge.^3^

The rise of generative artificial intelligence (AI) represents yet another wave of change.^4^ AI tools can assist with information retrieval, answering queries, offering recommendations and navigating resources.^4^ This combination of digital platforms and AI may once again disrupt the function and form of medical libraries. A final question lingers: What will medical libraries look like in the next 20 years?
